# 剧毒性Ⅱ型核糖体失活蛋白蓖麻毒素和相思子毒素的检测鉴定方法研究进展

**DOI:** 10.3724/SP.J.1123.2020.10001

**Published:** 2021-03-08

**Authors:** Longhui LIANG, Junmei XIA, Changcai LIU, Shilei LIU

**Affiliations:** 1.国民核生化灾害防护国家重点实验室, 北京 102205; 1. State Key Laboratory of NBC Protection for Civilian, Beijing 102205, China; 2.防化研究院分析化学实验室, 北京 102205; 2. The Laboratory of Analytical Chemistry, Research Institute of Chemical Defence, Beijing 102205, China

**Keywords:** Ⅱ型核糖体失活蛋白, *N*-糖苷酶活性, 毒素, 分析检测方法, 综述, type Ⅱ ribosome-inactivating proteins (RIPs), *N*-glycosidase activity, toxin, detection methods, review

## Abstract

Ⅱ型核糖体失活蛋白(RIPs)是一类重要的蛋白毒素,该类毒素大都具有一对二硫键连接的A-B链结构特征,B链具有半乳糖结合特性,能够与真核细胞膜表面受体特异性结合,将具有*N*-糖苷酶活性的A链导入细胞,与核糖体特定位点发生脱嘌呤作用使核糖体失活,最终通过抑制蛋白质合成而展现出细胞毒性。Ⅱ型RIPs毒素毒性极强,来源于植物的蓖麻毒素(ricin)和相思子毒素(abrin)的毒性分别是神经性毒剂维埃克斯(Vx)的385倍和2885倍。同时,该类毒素来源广泛、易于制备、稳定性好,成为一类潜在化生恐怖战剂,受到国内外广泛关注,其中蓖麻毒素作为唯一的蛋白毒素被收录于禁止化学武器公约禁控清单。近年来发生的多次蓖麻毒素邮件恐怖事件,进一步促进了有关Ⅱ型RIPs毒素的准确、灵敏、快速的检测鉴定技术的发展。剧毒性Ⅱ型RIPs毒素的检测鉴定方法主要涉及免疫分析法为代表的特异性识别和生物质谱分析为主的定性定量检测方法,以及基于脱嘌呤反应活性和细胞毒性的毒素活性检测方法。基于抗原-抗体作用的免疫检测法及基于寡核苷酸适配体的特异性识别检测法具有速度快、灵敏度高的优势,但对于复杂样品中高度同源蛋白的检测,易产生假阳性结果。随着生物质谱技术的快速发展,电喷雾离化(ESI)或基质辅助激光解吸离化(MALDI)等技术广泛应用于蛋白质的准确鉴定,不仅能够提供蛋白毒素的准确分子量和结构序列信息,而且能够实现准确定量。酶解质谱法是应用最为广泛的检测鉴定方法,通过酶解肽指纹谱分析,实现蛋白毒素的准确鉴定;对于复杂样品中蛋白毒素的分析,通过多种蛋白酶酶解策略获得丰富的特异性肽段标志物,然后进行肽段标志物的靶向质谱分析从而获得准确的定性及定量信息,方法有效提升了Ⅱ型RIPs毒素鉴定的准确度和灵敏度。免疫分析法和生物质谱法能够准确鉴定Ⅱ型RIPs毒素,但无法识别毒素是否还保持毒性。对于Ⅱ型RIPs毒素的活性分析,主要包括基于*N*-糖苷酶活性的脱嘌呤反应测定法和细胞毒性测定法,两种方法均可实现毒素毒性的简便、快速、灵敏的分析检测,是Ⅱ型RIPs毒素检测方法的有效补充。由于该类毒素的高度敏感性,国际禁止化学武器组织(OPCW)对相关样品中Ⅱ型RIPs毒素的分析提出了唯一性鉴定的技术要求。该文引用了Ⅱ型RIPs毒素及其检测方法相关的70篇文献,综述了以上Ⅱ型RIPs毒素的结构性质、中毒机理及典型剧毒性Ⅱ型RIPs毒素检测方法的研究进展,对不同检测方法的特点和应用潜力进行了总结,并结合OPCW对Ⅱ型RIPs毒素唯一性鉴定的技术需求,展望了未来Ⅱ型RIPs毒素检测技术研究的发展趋势。

## Abstract (en)

Type Ⅱ ribosome-inactivating proteins (RIPs) are an important class of protein toxins that consist of A and B chains linked by an interchain disulfide bond. The B-chain with lectin-like activity is responsible for binding to the galactose-containing receptors on eukaryotic cell surfaces, which is essential for A-chain internalization by endocytosis. The A-chain has *N*-glycosidase activity that irreversibly depurinates a specific adenine from 28S ribosomal RNA (28S rRNA) and terminates protein synthesis. The synergistic effect of the A-B chain inactivates the ribosome, inhibits protein synthesis, and exhibits high cytotoxicity. Ricin and abrin that are expressed by the plants *Ricinus communis* and *Abrus precatorius*, respectively, are typical type Ⅱ RIPs. The toxicity of ricin and abrin are 385 times and 2885 times, respectively, more that of the nerve agent VX. Owing to their ease of preparation, wide availability, and potential use as a bioterrorism agent, type Ⅱ RIPs have garnered increasing attention in recent years. Ricin is listed as a prohibited substance under schedule 1A of the Chemical Weapons Convention (CWC). The occurrence of ricin-related bioterrorism incidents in recent years has promoted the development of accurate, sensitive, and rapid detection and identification technology for type Ⅱ RIPs. Significant progress has been made in the study of toxicity mechanisms and detection methods of type Ⅱ RIPs, which primarily involve qualitative and quantitative analysis methods including immunological assays, mass spectrometry analysis methods, and toxin activity detection methods based on depurination and cytotoxicity. Immunoassays generally involve the specific recognition of antigens and antibodies, which is based on oligonucleotide molecular recognition elements called aptamers. These methods are fast and highly sensitive, but for highly homologous proteins in complex samples, they provide false positive results. With the rapid development of biological mass spectrometry detection technology, techniques such as electrospray ionization (ESI) and matrix-assisted laser desorption ionization (MALDI) are widely used in the identification of proteins. These methods not only provide accurate information on molecular weight and structure of proteins, but also demonstrate accurate quantification. Enzyme digestion combined with mass spectrometry is the predominantly used detection method. Accurate identification of protein toxins can be achieved by fingerprint analysis of enzymatically digested peptides. For analysis of protein toxins in complex samples, abundant peptide markers are obtained using a multi-enzyme digestion strategy. Targeted mass spectrometry analysis of peptide markers is used to obtain accurate qualitative and quantitative information, which effectively improves the accuracy and sensitivity of the identification of type Ⅱ RIP toxins. Although immunoassay and mass spectrometry detection methods can provide accurate identification of type Ⅱ RIPs, they cannot determine whether the toxins will retain potency. The widely used detection methods for activity analysis of type Ⅱ RIPs include depurination assay based on *N*-glycosidase activity and cytotoxicity assay. Both the methods provide simple, rapid, and sensitive analysis of type Ⅱ RIP toxicity, and complement other detection methods. Owing to the importance of type Ⅱ RIP toxins, the Organization for the Prohibition of Chemical Weapons (OPCW) has proposed clear technical requirements for the identification and analysis of relevant samples. We herein reviewed the structural characteristics, mechanism of action, and the development and application of type Ⅱ RIP detection methods; nearly 70 studies on type Ⅱ RIP toxins and their detection methods have been cited. In addition to the technical requirements of OPCW for the unambiguous identification of biotoxins, the trend of future development of type Ⅱ RIP-based detection technology has been explored.

核糖体失活蛋白(RIPs)是一类*N*-糖苷酶家族的特征毒蛋白,基于蛋白结构,RIPs被分为Ⅰ型、Ⅱ型、Ⅲ型共3类^[1]^。Ⅰ型RIPs由单亚基或两个亚基通过非肽键相连构成,相对分子质量约为30 kDa,具有RNA-*N*-糖苷酶活性,例如商陆植物(*Phytolacca acinosa*)的PAP蛋白和玉米黍的Maize b-32蛋白等^[2,3]^。Ⅲ型RIPs全部由单亚基构成,相对分子质量约为25 kDa,通常以前体形式存在,包括*N*-糖苷酶活性区和未知功能区,经酶切加工转换生成有活性的ⅡI型RIPs,目前,仅在大麦中分离检测出Ⅲ型RIP蛋白JIP60^[4]^。Ⅱ型RIPs是两个亚基通过二硫键连接构成的异二聚体蛋白,含有A、B链,其中A链为功能链,具*N*-糖苷酶活性;B链为结合链,具半乳糖结合的凝集素活性。与I型和Ⅲ型RIPs相比,Ⅱ型RIPs多了具有凝集素活性的B链,导致其毒性远高于I型和Ⅲ型RIPs^[3]^。目前,除在少数几种细菌和真菌中发现以外,Ⅱ型RIPs主要存在于一些有毒植物中,并且具有明显的组织特异性,普遍发现植物成熟种子中Ⅱ型RIPs活性相对较高^[2]^。3种类型的RIPs结构及代表蛋白质如图1所示。

**图1:**
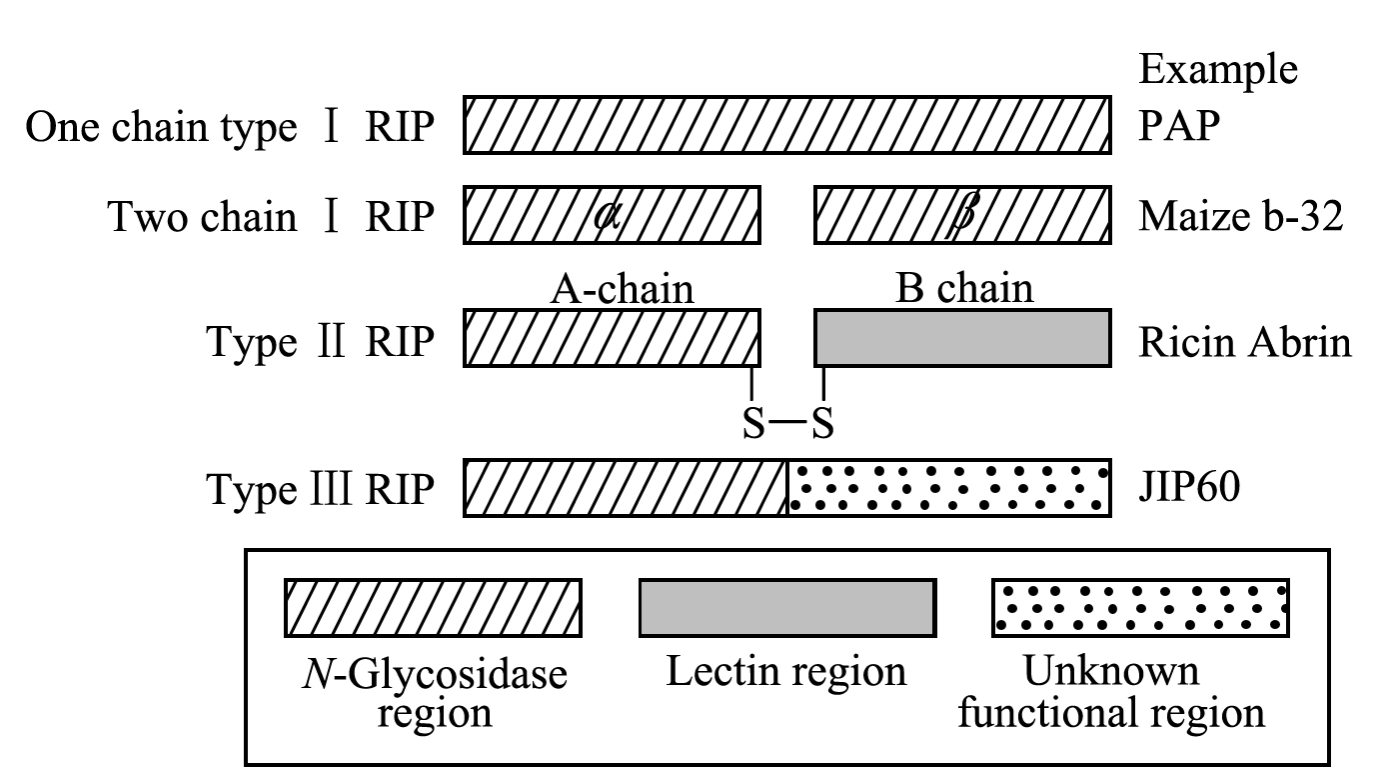
3种类型核糖体失活蛋白的结构示意图^[1]^

Ⅱ型RIPs发挥毒性作用的分子机理是能够特异、不可逆地催化脱去真核细胞核糖体的28S rRNA中保守GAGA-茎环结构中的第一个腺苷上的腺嘌呤,导致核糖体失去进行正常的蛋白质合成功能,从而引起细胞死亡^[5,6]^。表1列举了代表性剧毒Ⅱ型RIPs蛋白的来源及毒性^[2]^。在已发现的剧毒Ⅱ型RIPs毒素中,蓖麻毒素和相思子毒素的毒性最强、危害最大。由于分布广泛、易于获得、致命性强等特点,Ⅱ型RIPs被一些国家军方深入研究并制作为化学武器^[7]^,第一次世界大战期间,美军曾对蓖麻毒素开展过深入研究,并开展了蓖麻毒素作为吸入剂的测试试验。在第二次世界大战期间,英国军方研发了一种含有蓖麻毒素的复合炸弹,只是由于难以将大剂量的蓖麻毒素原体转化为气溶胶状态,这类武器还未在正式战争中使用^[4]^。但是,蓖麻毒素经常被作为生物恐怖战剂用于恐怖袭击以及政治暗杀等活动中^[7]^。1978年在伦敦的国际间谍人员曾用装有蓖麻毒素的伞尖在公开场所行刺,造成保加利亚作家及持不同政见者乔治·马可夫中毒身亡。2013年和2020年,美国时任总统奥巴马和特朗普都收到了含有蓖麻毒素粉末的信件。此外,Ⅱ型RIPs毒素来源广泛,易于制备,一旦被不法分子掌握其制备技术,将会成为严重的安全隐患。

**表1:** 剧毒Ⅱ型RIPs及其毒性^[2]^

Toxin	Source	Toxicity		
		Hela cell IC_50_/(mol/L)^a)^		Mice injection LD_50_/(μg/kg)^b)^
Abrin (相思子毒素)	*Abrus precatorius* seeds	3.9×10^-12^		0.04
Ricin (蓖麻毒素)	*Ricinus communis* seeds	6.0×10^-13^		3
Mistletoe lectin I (槲寄生凝集素I)	*Viscum album* leaves	1.7×10^-9^		2.4
Modeccin (药莲毒素)	*Adenia digitata* root	2.8×10^-12^		5.3
Volkensin (蒴莲毒素)	*Adenia volkensii* root	3.0×10^-13^		1.7
RIP (RIP毒素)	*Adenia goetzii* caudex	1.0×10^-12^		-
Lanceolin (柳杉毒素)	*Adenia lanceolata* caudex	5.0×10^-13^		6.8
Stenodactylin (西番莲毒素)	*Adenia stenodactyla* caudex	3.0×10^-13^		1.2
Aralin (阿拉林毒素)	*Aralia elata* shoots	1.3×10^-12^		-
Riproximin (利普西敏毒素)	*Ximenia americana* powder	1.1×10^-12^		-

a) IC_50_: half protein synthesis inhibitory concentration; b) LD_50_: lethal dose of 50%. -: not reported.

因此,无论是化学武器核查还是生物恐怖防御,对剧毒Ⅱ型RIPs毒素开展检测研究十分重要,也是国内外学者关注的热点。目前国际禁止化学武器组织(OPCW)正在组织国际相关领域实验室开展毒素分析演练工作,旨在形成技术体系完善的检测能力,并将毒素分析结果根据获得的信息分为4级,分别是:唯一性鉴定(unambiguous)、确证(confirmed)、临时性确证(provisional)和未确证(unconfirmed)。由于Ⅱ型RIPs毒素的高度敏感性,OPCW要求对样品必须实现最高级别的唯一性鉴定^[8]^,这包括必须提供:1)应用质谱或电泳检测的毒素分子量信息;2)应用酶解-串联质谱检测至少两条分别来源于A链和B链肽段标志物的质谱鉴定结果;3)应用酶联免疫吸附检测(ELISA)检测的毒素定量分析结果;4)应用脱嘌呤或体外细胞毒性检测方法对Ⅱ型RIPs毒素A链的脱嘌呤活性以及可选的B链凝集素活性的鉴定结果。本课题组所在的防化研究院分析化学实验室作为OPCW指定实验室代表中国持续参加该毒素分析演练任务,研究并构建了包括酶解质谱结构鉴定、ELISA定量检测以及体外脱嘌呤活性检测等Ⅱ型RIPs毒素唯一性鉴定技术体系^[9,10,11,12]^。本文综述了典型Ⅱ型RIPs蓖麻毒素和相思子毒素的理化性质、毒理作用及其检测鉴定方法研究进展,以期为开展相关科学研究提供参考依据。

## 1 Ⅱ型RIPs的毒性作用机理

### 1.1 毒理作用

Ⅱ型RIPs毒素的A链具*N*-糖苷酶活性,特异性水解真核细胞核糖体60S大亚基28S rRNA保守茎环结构顶部四核苷酸GA^4324^GA的腺嘌呤残基;Ⅱ型RIPs毒素的B链具凝集素特性,通过两个球状结构域发挥活性,每个结构域含有一个半乳糖结合位点,与细胞表面的半乳糖结合,介导A链进入细胞^[13]^,导致核糖体失活,从而抑制蛋白质合成,最终引起细胞死亡^[9,10,11]^。研究^[14]^显示,蓖麻毒素与HeLa细胞具较强的相互作用,在每个细胞上检测到约3.3×10^7^个毒素结合位点,结合常数达到2.6×10^7^ mol^-1^。然而,Ⅱ型RIPs对植物细胞展现的毒性远不如动物细胞,原因可能是植物细胞壁表面的半乳糖结合位点较少^[2]^。

Ⅱ型RIPs毒性强,相思子毒素和蓖麻毒素的小鼠静脉注射毒性是神经性毒剂维埃克斯(Vx)的2885倍和385倍。同时,研究^[15,16]^发现,序列高度同源的Ⅱ型RIPs毒素以及毒素不同亚型之间的毒性具有较大差异。例如,同样来源于植物蓖麻子中的蓖麻凝集素(RCA120)具有与蓖麻毒素相似的糖苷酶活性,其氨基酸序列与蓖麻毒素A链和B链的同源性分别达到了93%和84%,但其细胞毒性不足蓖麻毒素的十分之一^[17]^。相思子毒素存在a、b、c和d 4种亚型,同源性高达78%,但毒性差异较大,其中相思子毒素-b和相思子毒素-c由于其B链的凝集素活性较低而只有较弱的细胞毒性,反之相思子毒素-a与相思子毒素-d具有极强的细胞毒性^[15,18]^。RIPs细胞毒性产生巨大差异的原因主要与细胞表面的受体数量、受体亲和力以及RIPs本身的抗降解能力等因素有关。

### 1.2 细胞内转运途径

对于Ⅱ型RIPs毒素在细胞内的代谢途径,目前公认的机理是^[19]^: Ⅱ型RIPs毒素通过胞吞作用进入细胞膜,一部分返回细胞膜表面,另一部分进入初级内体(early endosomes)后被转运至次级内体(late endosomes);次级内体中的毒素大部分进入溶酶体中进行降解,仅约5%的毒素原体到达高尔基体反面网络结构,经逆向转运至内质网(ER);在蛋白质二硫键异构酶和分子伴侣的作用下,Ⅱ型RIPs毒素A链和B链解离,暴露出疏水区域的A链从ER释放到细胞质中发挥毒理作用(见图2)^[7]^。此A、B链解离代谢途径在酵母中得到了证实,研究^[20]^表明进入内质网的蓖麻毒素A链(RTA)通过误折叠激活内质网相关蛋白质降解途径(ERAD),将A、B链解离并使RTA穿过ER膜进入胞浆后在完整核糖体诱导下重新折叠,恢复活性,催化28S rRNA脱去腺嘌呤,破坏核糖体结构,导致细胞死亡。

**图2:**
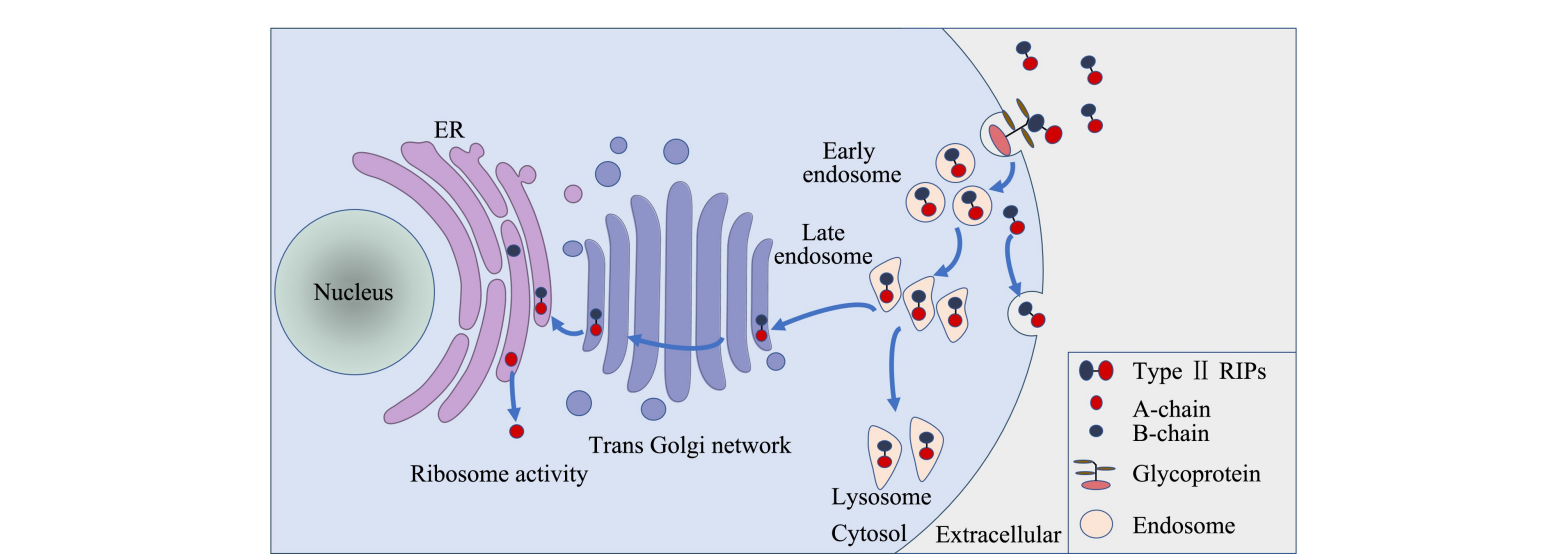
Ⅱ型RIPs毒素进入细胞及在细胞内运输途径的示意图^[7]^

## 2 Ⅱ型RIPs的检测鉴定方法

由于Ⅱ型RIPs毒素的特点及毒害作用,使得此类致命毒素在化生防护、化武履约核查和防反化生恐怖等工作中备受关注。利用分析检测技术准确鉴定环境及生物医学等样本中的Ⅱ型RIPs毒素,可为Ⅱ型RIPs毒素的使用取证、核查分析以及染毒人员的救治提供重要技术依据。基于结构与活性,我们将剧毒性Ⅱ型RIPs毒素的检测方法分为3大类,第一类是基于免疫等原理的特异性识别检测方法分析方法,第二类是生物质谱分析方法,前两类均属于毒素非活性定性定量分析方法;第三类是基于脱嘌呤反应活性和细胞毒性的毒素活性体外检测分析法。

### 2.1 特异性识别检测方法

2.1.1 酶联免疫吸附检测

ELISA是将可溶性的抗原或抗体结合到聚苯乙烯等固相载体上,利用抗原抗体专一性结合进行免疫反应的定性和定量检测方法。ELISA方法种类较多且具有敏感度高、特异性强、操作简便等特点,使其在Ⅱ型RIPs毒素的检测中应用最为广泛。随着增强比色法和化学发光技术的出现,进一步提高了ELISA方法的灵敏度^[21,22]^。Shyu等^[23]^建立了尿液和血清样品中蓖麻毒素的双抗体夹心ELISA定量检测方法,检出限达到了5.0 ng/mL。王晨宇等^[24]^利用蓖麻毒素单克隆抗体(MAb)3D74和标记的小鼠抗-蓖麻毒素单克隆抗体4C13辣根过氧化物酶(HRP),建立了定量检测血清和大鼠组织样品中蓖麻毒素的双夹心ELISA方法,检出限为1.25 ng/mL,方法应用于蓖麻毒素组织分布的定量测定,在静脉染毒大鼠的心、肝、脾、肺、肾、肠和脂肪等组织中均成功检测出蓖麻毒素。Leith等^[25]^应用亲和素-生物素-过氧化物酶复合物法(avidin-biotin-peroxidase complex method, ABC法),建立了动物组织样本中蓖麻毒素的竞争ELISA检测方法,检出限达到0.2 ng/mL。ELISA方法的缺点是对抗体的要求极为苛刻,一旦出现抗体对同源性抗原间的交叉反应,极容易产生假阳性结果。同源蛋白干扰(如蓖麻毒素与蓖麻凝集素RCA120,相思子毒素与相思子凝集素(abrus agglutinin))一直是ELISA方法检测Ⅱ型RIPs毒素的技术瓶颈,随着样品基质复杂程度的提高,出现假阳性结果的可能性也大大提高。为了区分Ⅱ型RIPs同源蛋白蓖麻毒素和RCA120,德国Robert Koch研究中心^[26]^开发了蓖麻毒素和RCA120的专属双夹心ELISA方法。蓖麻毒素-ELISA双抗体夹心方法应用单抗R18(抗蓖麻毒素A链)与单抗R109(抗蓖麻毒素B链),对蓖麻毒素的最低检出限达到2 pg/mL,定量范围为5~708 pg/mL,在蓖麻毒素定量线性检测范围内,该方法与同源蛋白RCA120基本不发生交叉反应;RCA120-ELISA双抗体夹心方法应用RCA120单抗ARK4和多抗IGY RC22,对RCA120的最低检出限达到1 pg/mL,定量范围为3.00~1549 pg/mL,在RCA120的线性检测范围内,该方法对同源蛋白蓖麻毒素基本不发生交叉反应。该中心^[27]^应用这两个方法,在2013年国际“复杂基质中蓖麻毒素定性和定量检测”能力考试中,准确鉴定出9个样品中添加的蓖麻毒素或蓖麻凝集素,定量检测结果准确度均达到90%以上。2017年,He等^[28]^从7种相思子毒素新型单克隆抗体中分别筛选出专属相思子毒素A链和B链的单克隆抗体,并建立了相思子毒素夹心ELISA检测方法,该方法能够区分相思子毒素和其同源蛋白相思子凝集素,对牛奶等基质,相思子毒素检出限达为1 ng/mL。随着抗体技术的不断发展,ELISA分析法在专属性方面取得了很大的进步,然而,要想获得专属性强的抗体,制备成本非常高,而且需要忍受较短的储存期限和苛刻的保存条件。

2.1.2 蛋白质免疫印迹法

蛋白质印迹法(western blot, WB)是将经过凝胶电泳分离的蛋白质样品转移到固相膜载体(例如硝酸纤维素薄膜)上,再利用抗原抗体结合专一性进行免疫反应的定性和定量检测方法。近年来,WB也常应用于Ⅱ型RIP毒素的检测,Lang等^[29]^采用抗RTA的兔血清作为抗体,建立了蓖麻毒素的WB检测方法,最低检出限可达1 ng。该方法已成功应用于兔血清、肝脏组织样品中蓖麻毒素的检测,能够分别在最低加标水平为0.67 μg/mL的兔血清、肝脏组织匀浆样本中检测出蓖麻毒素。

2.1.3 免疫磁性微球及生物传感检测

免疫磁性微球(immunomagnetic microspheres, IMMS)检测将目标蛋白抗体与磁性微球进行偶联,特异性捕获复杂基质中的目标蛋白,从而达到分离纯化和检测目的。免疫磁性微球捕获具分离速度快、效率高、操作简单、重现性好等优点,结合荧光、化学发光或电化学发光检测技术,极大地提升了检测速率,使检测时间由8 h缩短至2~4 h^[30,31]^。基于免疫原理发展的生物传感检测技术,其最大优点是能够通过微型芯片的设计,实现多种毒素的同时快速检测,是事故现场快速检测的有效手段。Delehanty等^[32]^使用配备电荷耦合原件的抗体微阵列免疫芯片,在15 min内实现了霍乱毒素、葡萄球菌肠毒素B(SEB)、蓖麻毒素和芽孢杆菌的同时检测,其中蓖麻毒素的检出限为10 ng/mL。Ligler等^[33]^对免疫芯片进一步优化设计,实现了6种毒素的同时检测。Mu等^[34]^发展了一种基于隧道磁阻(TMR)生物传感器的蓖麻毒素快速检测新方法,将磁免疫色谱试纸与TMR磁敏传感器信号检测有效地结合在一起,通过检测功能化磁信号探针在免疫色谱试纸上产生的磁场强度,实现蓖麻毒素的快速检测,定量检测的线性范围为1 ng/mL~200 μg/mL。该方法克服了复杂环境干扰物对毒素检测的影响,可以满足水、土壤、食物、血液等复杂样品的分析要求。Nasirahmadi等^[35]^以含有9个氨基酸的肽段分子印迹聚合物作为模板,在紫外光作用下,在功能单体和表位之间的氢键作用下形成分子印迹聚合物,然后使用2.5%的十二烷基硫酸钠(SDS)和0.6%乙酸对聚合物进行衍生,最终得到生物传感芯片;将设计的纳米生物传感器应用于血浆和尿样等样品中槲寄生蛋白毒素的快速检测,检出限分别为0.5和1.25 ng/μL。

2.1.4 适配体分析法

目前已报道的基于抗体识别原理的免疫分析法具有灵敏度高、专属性好、耗时短、定量准确等优点,广泛应用于复杂基质中Ⅱ型RIPs蛋白蓖麻毒素和相思子毒素的检测。存在的不足是:方法依赖亲和力强、效价高的抗体,对实验室抗体制备能力要求较高,且抗体的贮存稳定性较差。Shu等^[36]^通过指数富集的配体系统进化技术(SELEX),结合PCR体外扩增,经过数轮反复的体外筛选、扩增得到与蓖麻毒素抗体亲和力和特异性相当的寡核苷酸适配体,建立了基于适配体识别的多纳米孔检测技术,实现了蓖麻毒素的有效检测。Tang等^[37,38]^使用SELEX技术筛选出了蓖麻毒素和相思子毒素的适配体并应用于两种毒素的定量检测,定量限分别为1 μg和0.3 μg。与免疫识别法相比,适配体的最大优势在于稳定性好、可反复使用,但存在筛选过程复杂、灵敏度较差等问题。因此Ⅱ型RIPs毒素的特异性识别技术的一个关键在于设计制备稳定性好、灵敏度高的适配体。

### 2.2 生物质谱分析

20世纪80年代发展起来的基质辅助激光解吸附离子化(MALDI)和电喷雾离子化(ESI)技术,为分析稳定性差、难挥发的生物大分子样品提供了优越的分析手段,越来越多的科学家将质谱技术应用于核酸、蛋白质等大分子的检测与鉴定。如今,生物质谱法已成为复杂样品中蛋白质鉴定的基本方法。利用生物质谱技术可以实现大分子蛋白质相对分子质量的测定,2000年,Despeyroux等^[39]^使用ESI-四极杆(Q)质谱对蓖麻毒素进行鉴定,蓖麻毒素原体经电喷雾离化产生多电荷离子,将数据进行解卷积计算,最终得到平均相对分子质量为63 kD的质谱峰簇,即为蓖麻毒素的相对分子质量。高分辨质谱的发展可以提供更为准确的相对分子质量信息,从而有效提升鉴定结果准确性。例如MALDI-飞行时间(TOF)高分辨质谱被报道用于鉴定完整蓖麻毒素原体,该方法检测到的蓖麻毒素的相对分子质量为62766 Da^[40]^。然而,在实际应用中,相对分子质量测定法仍无法给出Ⅱ型RIPs毒素的准确结构信息^[41]^。随后,人们^[42]^尝试在分析前将毒素进行酶解,再应用LC-MS分析肽指纹图谱或LC-MS/MS靶向测定特异性肽段,通过酶解肽段氨基酸序列的鉴定,鉴定蛋白毒素的序列结构,依据这种策略发展出了酶解质谱分析法。

2.2.1 酶解质谱鉴定法

与免疫分析方法相比,酶解质谱法具有很多优势:1)能够准确鉴定毒素特异性肽段的氨基酸序列,从而实现高度同源蛋白间的差异性鉴定;2)准确识别毒素蛋白分子内的二硫键位置及翻译后修饰信息;3)通过靶向特异性肽段的定量检测从而实现Ⅱ型RIPs毒素原体的定量检测。此外,酶解质谱技术可以与各种分离纯化技术(如凝胶亲和、免疫磁珠、一维及二维电泳等)结合,在质谱分析前对目标蛋白进行富集纯化,降低基质干扰,实现复杂基质中Ⅱ型RIPs毒素专一、灵敏的检测。酶解质谱法的分析策略如图3所示^[42]^。

**图3:**
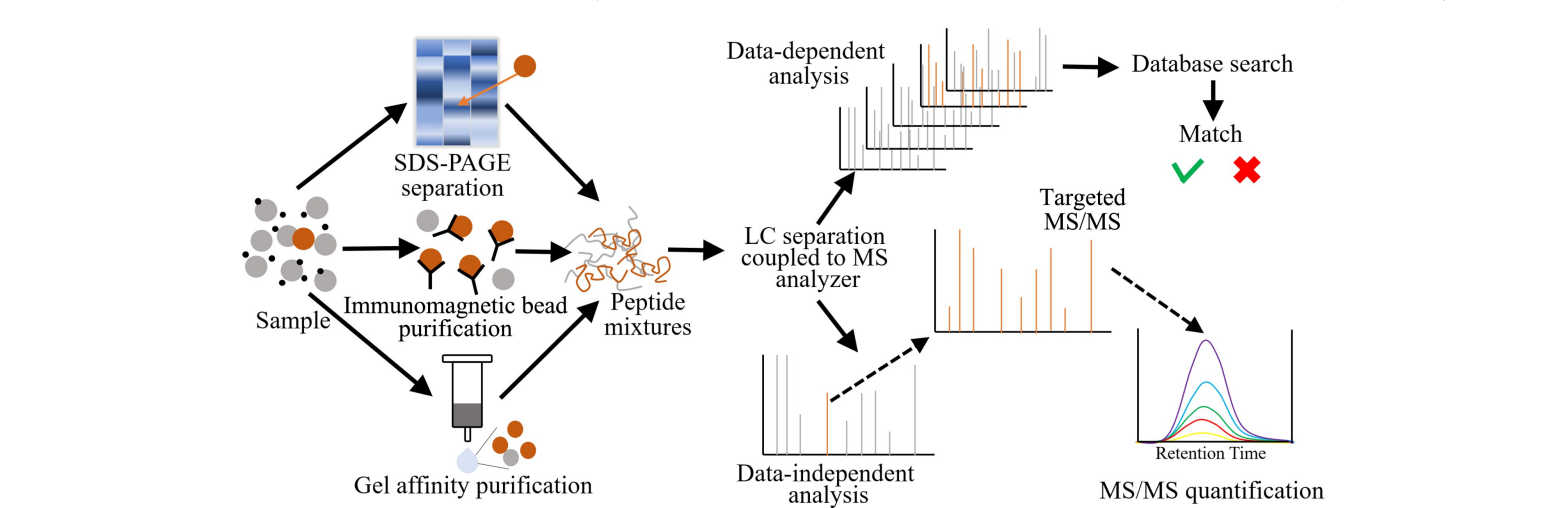
酶解质谱法分析蛋白策略^[42]^

酶解质谱法将大分子蛋白质酶解生成多肽混合物,然后使用高分辨质谱技术(LC-Q-TOF MS或MALDI-TOF MS)分析肽指纹图谱(PMF)或LC-ESI-MS/MS靶向测定特异性肽段,从而实现蛋白质的鉴定。2001年,Darby^[40]^首次将PMF法应用于蓖麻毒素的鉴定,分别使用LC-Q-TOF MS和MALDI-TOF MS对蓖麻毒素的酶解产物进行分析,结合数据库检索,成功鉴定出蓖麻毒素的14条肽段。Sousa等^[43]^采用加速溶剂萃取(ASE)技术处理含有蓖麻毒素的复杂样品,经SDS-聚丙烯酰胺凝胶(PAGE)、胰蛋白酶解后采用MALDI-TOF MS对酶解的肽段进行鉴定,通过PMF数据库检索,鉴定出蓖麻毒素的19条肽段,然后对其中3条唯一性肽段进行MALDI-TOF MS/MS二级质谱检测,实现了蓖麻毒素的唯一性鉴定。

这些方法虽然准确度高,但在酶解前需要变性、还原和烷基化等步骤,整个过程步骤多、周期长。为解决这一问题,有机溶剂辅助直接酶解蛋白质原体的方法被开发并应用于蓖麻毒素的分析鉴定,例如在酶解样品中使用甲醇等能够有效促进胰蛋白酶解效率、缩短酶解时间,这些方法在蓖麻毒素的鉴定与高灵敏检测等方面都取得了较好的结果。目前,T7A、T11A、T6B和T18B是蓖麻毒素的胰蛋白酶解肽段中使用最多的代表性肽段标识物,被广泛应用于粉末、蓖麻子粗提物、牛奶、饮料等复杂基质中痕量蓖麻毒素的唯一性鉴定^[41,44-49]^。但是,该方法存在酶解效率较低、易发生漏切等缺点。本课题组^[11]^研究发现乙腈有助于胰蛋白酶准确酶解毒素原体、高效生成标识性肽段,并开发和建立了一种乙腈辅助胰蛋白酶直接酶解蓖麻毒素原体的方法,显著提高了酶解效率,将酶解反应时间从18 h减至4 h。

随着技术的不断发展,近年来,免疫磁珠、凝胶亲和等技术常作为高效的样品制备手段被应用于复杂基质中目标物的分离与纯化,与质谱等技术相结合,在蛋白质鉴定等方面发挥着重要的作用。2011年,美国疾控中心(CDC)的McGrath等^[46]^通过制备修饰蓖麻毒素抗体的免疫磁珠,对饮用水、牛奶、果汁等基质中的蓖麻毒素进行富集,经胰蛋白酶酶解,利用液相色谱-线性离子阱质谱靶向鉴定蓖麻毒素A链和B链的标志性肽段(T7A和T18B),最终实现了蓖麻毒素的高灵敏鉴定与定量分析,最低检出限达到0.64 ng/mL。2015年,Fredriksson等^[50]^采用半乳糖凝胶树脂填充柱,对饮用水,饮料、粉末以及擦拭样品中的典型剧毒Ⅱ型RIPs毒素(蓖麻毒素、相思子毒素、蓖麻凝集素)进行选择性富集,结合胰蛋白酶解和高分辨质谱靶向检测技术,实现了复杂样品中Ⅱ型RIPs毒素准确鉴定。Feldberg等^[51]^采用琼脂糖凝胶亲和富集结合三重四极杆串联质谱技术建立了复杂环境样品中痕量蓖麻毒素的检测方法;该方法对从不同地理区域获取的60种不同环境样本(例如土壤、沥青和植被)进行测定,蓖麻毒素的最低检出限达到1 ng/mL(g)。

除了使用胰蛋白酶解以外,近年来,科学工作者们还探索了其他酶解方法。本课题组^[12]^系统研究了蓖麻毒素在不同蛋白酶解(糜蛋白酶、胃蛋白酶、蛋白酶K和胰蛋白酶/胞内蛋白酶(Glu-C)串联酶解)体系中的最优酶解条件,采用LC-ESI-Q/TOF MS高分辨质谱对酶解产生的肽段进行鉴定,结合美国国家生物技术信息中心(NCBI)数据库检索比对,对所有肽段的专属性进行确认与分类,最终筛选鉴定出专属性强且质谱响应好的肽段作为蓖麻毒素的肽段标志物,大大丰富了唯一性鉴定蓖麻毒素肽段标志物的选择范围。此外,本课题组^[52]^通过离子交换、HPLC等方法对我国东北槲寄生植物(*Viscum. Oloratum* Nakai)中的槲寄生毒素进行分离纯化,使用Glu-C酶解槲寄生毒素,然后采用MALDI-TOF MS对酶解的肽段进行测定,首次鉴定出含有四对二硫键的槲寄生毒素B7。Braun等^[53]^报道了使用糜蛋白酶蓖麻毒素进行酶解过夜,使用高分辨液相色谱-质谱对糜蛋白酶酶解产生的两条蓖麻毒素肽段进行靶向鉴定,成功实现了植物提取物和土壤样品中蓖麻毒素的准确鉴定。

近年来,酸消解技术也被用于Ⅱ型RIPs毒素的分析鉴定。Chen等^[54]^采用微波辅助-热酸消解方法选择性水解蓖麻毒素氨基酸序列中的天冬氨酸残基,水解时间仅需要15 min,大大提高了反应效率,结合MALDI-TOF MS检测,成功鉴定出蓖麻毒素中的二硫键肽段,为蓖麻毒素的物证鉴定提供了一种可靠的技术手段。

2.2.2 质谱定量检测

随着LC-MS技术的发展,目前除了实现痕量毒素的定性检测外,更多的努力被放在定量分析研究中。液相色谱-三重四极杆质谱(LC- QQQ MS)的多反应监测模式(MRM)和液相色谱-四极杆-轨道离子阱质谱(LC-Q-Orbitrap MS)的平行反应监测模式(PRM)在较宽的动态范围内具有很好的线性响应,在Ⅱ型RIPs毒素的定量分析方面得到了广泛的关注和应用。质谱定量离不开同位素标记的内标,目前的研究大多基于同位素标记多肽为内标的绝对定量策略(absolute quantitation peptide strategy, AQUA):在质谱分析前,向样品中添加已知浓度的同位素标记的多肽内标,由于内标肽段与目标肽段标识物具有相同的色谱和质谱特征,通过比较定量肽段和内标肽段的绝对丰度,实现复杂样品中目标蛋白质的定量检测。Schieltz等^[49]^采用AQUA方法和酶解质谱策略,对18种不同产地植物蓖麻子中的蓖麻毒素和RCA120进行定量检测,该研究揭示了不同产地蓖麻子中两种Ⅱ型RIPs毒素的含量差异信息。McGrath等^[46]^以同位素内标肽段建立了饮用水、牛奶、果汁等食源性基质中蓖麻毒素的免疫捕获-酶解质谱定量检测方法,线性范围在10~10000 fmol/mL。2017年,Hansbauer等^[18]^应用胰蛋白酶解结合液质检测不同亚型相思子毒素肽段标志物,首次实现了不同亚型相思子毒素和相思子总蛋白的定量检测。

AQUA定量方法虽然显著提高了复杂样品中目标蛋白质的定量准确度,但是由于内标肽段无法兼容复杂样品的前处理程序,只能在样品制备后加入内标,导致定量结果仍然存在偏差。针对以上问题,科学家^[55,56]^提出了以稳定同位素标记的蛋白质为内标的蛋白质内标绝对定量(protein standard absolute quantification, PSAQ)策略。基于无细胞快速表达系统,在体外表达合成稳定同位素标记的内标蛋白质,由于PSAQ内标与目标蛋白质具有相近的生化性质,因此可以在分析的最初阶段将蛋白质标样加入样品中,在样品制备及酶解过程中发生的蛋白质损失将不会影响蛋白质定量的准确性。Dupré等^[57]^使用单链PSAQ内标标记法结合Q-Orbitrap高分辨质谱定量研究体液、食品等复杂基质中的蓖麻毒素A链、金黄葡萄球菌毒素B和产气荚膜梭菌毒素(clostridium perfringens)3种毒素,检出限均达到1 ng/mL。该方法的主要是缺点是PSAQ内标的合成制备难度大、成本高昂,仅适用于Ⅱ型RIPs毒素单链的定量检测。全毒素内标的设计与制备,将是实现Ⅱ型RIPs毒素绝对定量检测的关键,然而,目前应用无细胞体外表达和细胞表达技术均未实现具链间二硫键的Ⅱ型RIPs全蛋白毒素同位素内标的生物合成。

### 2.3 毒性活性的体外检测

免疫分析法和酶解质谱法成功应用于Ⅱ型RIPs毒素的定性定量检测,但无法鉴定毒素是否还保持毒性。Ⅱ型RIPs毒素的活性鉴定在反应机理评估、酶学性质研究、寻找抑制剂以及毒素的检测诊断等方面具有重要意义。由于该类毒素的高度敏感性,禁止化学武器组织在核查分析中,不仅要求对其进行定性定量检测,同时还要求对其活性进行准确表征。目前,对于Ⅱ型RIPs毒素的活性鉴定一般选择脱嘌呤活性的间接测定法和细胞毒性测定法。

2.3.1 脱嘌呤活性体外检测

1987年Endo等^[58]^开发的苯胺裂解测定法是最早用于检测Ⅱ型RIPs毒素的脱嘌呤活性的分析方法;结果表明,28S rRNA经Ⅱ型RIPs处理后迁移率发生变化,对核糖核酸酶产生了抗性,实验进一步证明,Ⅱ型RIPs以水解方式剪切了28S rRNA中A^4324^位的*N*-糖苷键;脱去A^4324^后,28S rRNA的第50和30个磷酸二酯键变得不稳定,聚丙烯酰胺凝胶染色结果显示,苯胺处理后释放出约400个核苷酸的RNA片段,实现蓖麻毒素的活性鉴定。

2010年,Melchior等^[59]^开发了一种定量实时PCR检测方法,该方法利用逆转录酶将一个腺嘌呤插入模板rRNA中发生脱嘌呤反应的碱基位点上,使该位点从T转化为A;在55 ℃、pH 5.0条件下,蓖麻毒素与模板RNA相互作用,然后反转录为cDNA;使用定量PCR法对突变和未突变的RNA进行定量检测,实现蓖麻毒素对RNA底物的脱嘌呤活性鉴定。该方法被应用于检测环境样品和食品基质中的低浓度蓖麻毒素的脱嘌呤活性,方法灵敏度高、重复性好。

1989年,Zamboni等^[60]^首次使用HPLC检测到真核生物核糖体经脱嘌呤反应释放的腺嘌呤:将蓖麻毒素等Ⅱ型RIPs蛋白与真核生物核糖体进行孵育反应,经乙醇提取、氯乙醛衍生,最终使用荧光检测器通过HPLC检测腺嘌呤,氯乙醛衍生大大提高了HPLC对腺嘌呤的检测灵敏度,蓖麻毒素的检测限为100 ng。后来的研究发现,Ⅱ型RIPs毒素还可以对DNA、RNA及poly(A)底物发生脱嘌呤作用^[61,62]^。Chen等^[63]^采用HPLC方法研究RTA对茎环结构的RNA底物脱嘌呤反应机理及动力学,结果表明,RTA对RNA底物的催化速率在pH 4.0条件下达到最高。HPLC分析法的缺点是,氯乙醛衍生物必须进一步用饱和的乙醚萃取,并通过0.45 μm过滤等操作才能用于HPLC分析,导致该方法步骤复杂,耗时较长。

为了避免HPLC分析法操作繁琐的衍生步骤,质谱、表面增强拉曼光谱(SERS)等技术被引入,用于毒素的脱嘌呤活性检测。Becher等^[64]^将抗体磁珠富集后的蓖麻毒素与人工合成的RNA底物在体外特定的条件下孵育,使用LC-MS检测样品中的腺嘌呤,对蓖麻毒素的最低检出限达到0.1 ng/mL。Suzanne等^[65]^应用抗体磁珠富集纯化复杂样品中的蓖麻毒素,然后与人工合成的单链DNA底物进行体外脱嘌呤反应,最后利用MALDI-TOF/MS检测DNA底物在反应前后相对分子质量的变化,实现样品中活性蓖麻毒素的鉴定。Tang等^[66]^提出了一种基于SERS检测的蓖麻毒素活性检测方法,将连接单链DNA底物多聚A(21dA)的金纳米探针组装在二氢硫辛酸修饰的硅晶片(SH-Si)上,组成SERS芯片,蓖麻毒素与SERS芯片进行反应,通过监测脱嘌呤反应引起的腺嘌呤SERS信号的衰减实现活性蓖麻毒素的快速检测。最近,研究人员^[67]^还发展了脱嘌呤活性分析与荧光纳米探针技术的联用分析方法,并应用于复杂基质中活性Ⅱ型RIPs毒素的现场检测。

脱嘌呤分析法成功实现了痕量Ⅱ型RIPs毒素的活性鉴定,但是,此类方法使用的脱嘌呤DNA及RNA底物在酸性条件下都会发生不同程度的自水解反应,阴性对照具有较高的响应,导致方法灵敏度降低。目前在实际样品检测中,一般以高于10倍阴性对照响应作为检出标准。通过底物序列的设计优化,筛选出不易自水解的最适底物以提高检测方法的灵敏度和专属性,是此类方法迫切需要解决的技术难题和未来的发展趋势。

2.3.2 体外细胞毒性检测

对于Ⅱ型RIPs毒素的活性,常采用基于实验动物和细胞的毒性分析法。董娜等^[68]^采用3-(4,5-二甲基噻唑-2)-2,5-二苯基四氮bai唑溴盐(MTT)比色法探索了蓖麻毒素对不同细胞以及小鼠各主要组织脏器的毒性作用;细胞活性检测结果表明,不同浓度的蓖麻毒素对Caco-2、MDCK、MDCK-MDR1、HepG2和H1299细胞均有一定的毒性,毒素浓度与细胞毒性呈现明显的效量关系,MDCK细胞对蓖麻毒素最为敏感;病理学检测结果表明,在经灌胃方式中毒的小鼠血清中发现谷丙转氨酶(ALT)、谷草转氨酶(AST)、碱性磷酸酶(ALP)、总胆红素(TBIL)、甘油三酯(TG)、肌酐(CREA)均有升高趋势,表明小鼠中毒3 h内肝功能和肾功能已出现一定程度的损伤。

2005年,Zhao等^[69]^开发了一种基于萤光素酶表达的非放射性测定方法,使用半衰期短的编码荧光素酶不稳定衍生物的cDNA转染细胞;荧光素酶cDNA通过腺病毒转导进入细胞中,并利用荧光素酶的光输出来测量蛋白质的合成水平,从而实现Ⅱ型RIPs毒素的毒性测定。此外,该团队^[69]^还开发出一种基于96和384孔的高通量筛选方法,用于检测不同细胞对Ⅱ型RIPs毒素的敏感性;使用此分析方法筛选了针对Ⅱ型RIPs毒素的14000个小分子文库,发现两种化合物在Ⅱ型RIPs毒素的细胞内转运步骤中起抑制作用,该方法证明了使用非放射性分子筛选Ⅱ型RIPs毒素抑制剂的可行性。Wahome等^[70]^在2010年开发了一种简化的细胞荧光素酶测定法,该方法不需要用荧光素酶cDNA转染细胞,将Vero细胞接种在384孔板中,孵育过夜,加入萤光素酶以及萤光素底物,随后进行蓖麻毒素处理;萤光素酶的荧光信号强度与细胞ATP水平成正比,从而可以反映细胞的活力水平。该方法用于Ⅱ型RIPs毒素的活性测试灵敏度和信噪比均较高,并且具有较好的稳定性和重现性。

综上,虽然脱嘌呤分析法和体外细胞毒性检测方法能够实现Ⅱ型RIPs毒素的活性鉴定,但是目前此类方法无法区分Ⅱ型RIPs毒素的种类,需要结合酶解质谱等分析方法才能实现Ⅱ型RIPs毒素的唯一性鉴定。

## 3 结论与展望

由于Ⅱ型RIPs毒素具有毒性极强、制备简便以及难以防治的特点,其快速、准确的识别检测技术已成为各国反化生恐怖发展规划中的重要工作。然而,针对Ⅱ型RIPs毒素的检测方法各有优缺点,尚未有一种分析方法能够同时实现Ⅱ型RIPs毒素的准确鉴定与活性检测。正如OPCW对生物毒素唯一性鉴定的要求标准,实现剧毒Ⅱ型RIPs毒素(蓖麻毒素,相思子毒素等)的唯一性鉴定必须要同时准确获得其结构鉴定信息、毒素与抗体的免疫识别信息以及毒性活性3个方面的完整信息。因此,在实际样品的准确分析中,往往需要多种检测方法的联合使用,发展剧毒性Ⅱ型RIPs毒素的准确、灵敏、特异的多重检测方法是当前乃至今后生物毒素唯一性鉴定研究工作的重点。
